# Fresh Carrier for an Old Topical Local Anesthetic: Benzocaine in Nanostructured Lipid Carriers

**DOI:** 10.1021/acsbiomaterials.4c00585

**Published:** 2024-07-29

**Authors:** A. D. Souza, G. H. Rodrigues da Silva, L.N.M. Ribeiro, H. Mitsutake, H. N. Bordallo, M. C. Breitkreitz, P. C. Lima Fernandes, L. D. Moura, F. Yokaichiya, M. Franco, E. de Paula

**Affiliations:** † Departamento de Bioquímica e Biologia Tecidual, Instituto de Biologia, Universidade Estadual de Campinas (Unicamp), ZIP 13083-862 Campinas, São Paulo, Brazil; ‡ Laboratório Nacional de Biociências, Centro Nacional de Pesquisa em Energia e Materiais, ZIP 13083-100 Campinas, São Paulo, Brazil; § Niels Bohr Institute, University of Copenhagen, ZIP 2100 Copenhagen, Denmark; ∥ Departamento de Química Analítica, Instituto de Química, Unicamp, ZIP 13083-862 Campinas, São Paulo, Brazil; ⊥ Departamento de Física, Universidade Federal do Paraná (UFPR), ZIP 81531-980 Curitiba, Paraná, Brazil; # Instituto de Pesquisas Energéticas e Nucleares, IPEN-CNEN/SP, ZIP 05508-000 São Paulo, São Paulo, Brazil

**Keywords:** benzocaine, nanostructured lipid carriers, local anesthetic, experimental design, drug delivery

## Abstract

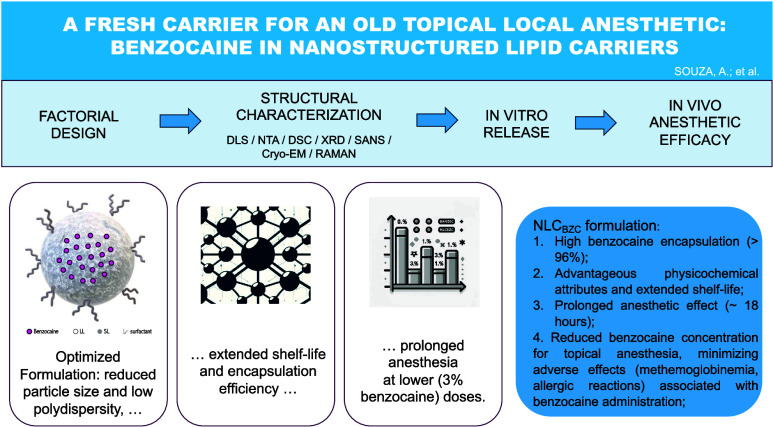

Nanostructured lipid carriers (NLC) have emerged as innovative drug delivery systems, offering distinct advantages over other lipid-based carriers, such as liposomes and solid lipid nanoparticles. Benzocaine (BZC), the oldest topical local anesthetic in use, undergoes metabolism by pseudocholinesterase, leading to the formation of *p*-aminobenzoic acid, a causative agent for allergic reactions associated with prolonged BZC usage. In order to mitigate adverse effects and enhance bioavailability, BZC was encapsulated within NLC. Utilizing a 2^3^ factorial design, formulations comprising cetyl palmitate (solid lipid), propylene glycol monocaprylate (liquid lipid), and Pluronic F68 as surfactants were systematically prepared, with variations in the solid/liquid lipid mass ratios (60:40–80:20%), total lipid contents (15–25%), and BZC concentrations (1–3%). The optimized formulation underwent characterization by dynamic light scattering, differential scanning calorimetry, Raman imaging, X-ray diffraction, small-angle neutron scattering, nanotracking analysis, and transmission electron microscopy (TEM)/cryo-TEM, providing insights into the nanoparticle structure and the incorporation of BZC into its lipid matrix. NLC_BZC_ exhibited a noteworthy encapsulation efficiency (%EE = 96%) and a 1 year stability when stored at 25 °C. In vitro kinetic studies and in vivo antinociceptive tests conducted in mice revealed that NLC_BZC_ effectively sustained drug release for over 20 h and prolonged the anesthetic effect of BZC for up to 18 h. We therefore propose the use of NLC_BZC_ to diminish the effective anesthetic concentration of benzocaine (from 20 to 3% or less), thus minimizing allergic reactions that follow the topical administration of this anesthetic and, potentially, paving the way for new routes of BZC administration in pain management.

## 1 Introduction

The International Association for the Study of Pain (IASP) defines pain as “an unpleasant sensory and emotional experience associated with, or resembling that associated with, actual or potential tissue damage”,^1^ which is expressed by organic reactions that vary from person to person, influenced by biological, psychological, and social factors. Local anesthetics (LAs) are largely used for pain control, either in surgical (infiltrative route) or in minor invasive procedures (mainly topical anesthesia). Benzocaine (BZC) is an ester-type compound that, in contrast to other LAs, consistently maintains an uncharged state at physiological pH; this results in a log *P* octanol–water of 1.9 and low (4.4 mM) water solubility,^2^ thereby limiting its systemic absorption and confining its application solely to topical use. BZC is the oldest topical anesthetic agent still in use, and in dental clinics, 20% of BZC gel is the most used topical LA with a fast onset of action, excellent superficial analgesia, and short duration.^3^ However, the prolonged use of BZC can induce allergic reactions due to *p*-aminobenzoic acid, a result from the action of (ester bond hydrolysis) pseudocholinesterases.^3,4^ Although serious toxicity with BZC is rare,^5^ the use of benzocaine products to treat mouth pain has increased attention for signs and symptoms of methemoglobinemia such as pale, gray, or blue-colored skin, lips, and nail beds; shortness of breath; fatigue; confusion; headache; lightheadedness, and fast heart rate, deserving an FDA Drug Safety Communication.^6^

The development of innovative drug delivery systems (DDS), able to carry and keep the LA at the site of application, and slowing down their systemic metabolization are ways to improve their bioavailability and reduce such adverse effects. Lipid-based DDS such as nanostructured lipid carriers (NLC) show many advantages for the encapsulation of LA, including high upload capacity, shelf stability, low cost, and safety,^7^ which make them very attractive for the pharmaceutical industry. The literature reports the successful use of NLC to improve the therapeutic activity of diverse LA agents, delaying their systemic degradation and providing sustained release.^8−17^

NLC is composed of a mixture of solid and liquid lipids that self-assemble into nanosized particles. This unique structure allows for the encapsulation of hydrophobic drugs, such as LA, improving their solubility and chemical stability. Additionally, the small size of NLC enables them to penetrate the stratum corneum, the outermost layer of the skin, more effectively than conventional DDS. This enhanced penetration can improve the delivery of benzocaine to the underlying tissues, resulting in a faster onset of action and prolonged pain relief. Furthermore, NLC can be tailored to release the encapsulated drug in a controlled manner, allowing for sustained release and a prolonged therapeutic effect. This is particularly advantageous for local anesthetics, which typically have a short duration of action and require repeated administration to keep antinociception. By incorporating LA into NLC, the frequency of application can be reduced, improving patient compliance and overall treatment outcomes. In addition to improving drug delivery, NLC offers other advantages for topical applications of LA: while protecting the encapsulated drug from degradation and metabolism, NLC increases drug bioavailability and efficacy. Furthermore, the lipid excipients of NLC can provide long shelf life stability to the formulation, reducing the need for preservatives and other additives that may cause skin irritation or allergic reactions. Overall, the use of NLC for the topical delivery of LA is a promising approach to improving pain management in various clinical settings. By enhancing the penetration, retention, and release of local anesthetics, NLC has the potential to increase the efficacy and safety of topical anesthesia, leading to better patient outcomes and overall satisfaction.

To fully explore the potential of NLC as a DDS for local anesthetics, we describe here the preparation by design of experiments (DoE), of a DDS engineered to effectively sustain the release and prolong the anesthetic effect of benzocaine, a quintessential topical LA. The optimized formulation efficiently encapsulated BZC in its lipid core and reduced the necessary dosage for effective anesthesia (from 20 to 3%), mitigating the occurrence of possible allergic reactions associated with its topical administration.

## 2 Materials and Methods

Benzocaine and the nonionic surfactant Pluronic F68 (P68) were purchased from Sigma-Aldrich (USA). The lipids cetyl palmitate (CP) and propylene glycol monocaprylate (Capmul PG8-NF) were supplied by Dhaymers Quím. Fina (Brazil) and Abitec Corp. (USA), respectively. All other reagents were of analytical grade.

### 2.1 Preparation of Nanostructured Lipid Carriers

NLC formulations were prepared following the emulsification–ultrasonication method.^18^ Briefly, the solid lipid (SL) cetyl palmitate was heated to 65 °C, above its melting point, followed by the addition of liquid lipid (LL) propylene glycol monocaprylate. After that, BZC was added to the oil phase until its complete dissolution. An aqueous phase of P68 in deionized water was heated to the same temperature, and both phases were blended under a high-speed agitation (3000 rpm) for 3 min with an Ultra-Turrax blender (IKA WerkeStaufen, Germany). The mixture was then sonicated for 30 min using a Vibracell tip sonicator (Sonics & Mat. Inc., Danbury, USA) operated at 60 W and 20 kHz in alternating 30 s, on–off cycles. Finally, the nanoemulsion was cooled down to room temperature in an ice bath to form the NLC.

#### 2.1.1 Design of Experiments

A 2^3^ factorial design was performed, with triplicate of the central points. The Design Expert software (Stat-Ease Inc., Minneapolis) version 12.0.1.0 was used to optimize the NLC formulations. Analysis of variance (ANOVA) was applied to verify the adjusted model, considered significant for *p*-value < 0.05.^19^ The independent variable levels and desired responses are given in Table 1. The amount of P68 was kept the same (5%) in all formulations.

**Table 1:** 2^3^ Factorial Design of NLC Formulations: Independent Variables, Their Levels, and Desired Responses^a^

experimental variables	low level	high level	central point
TL (m)	15%	25%	20%
SL/LL ratio (% w/w)	60:40	80:20	70:30
BZC (% w/w)	1	3	2

responses	goal
particle size (nm)	minimum
polydispersity index (PDI)	minimum
ZP (|mV|)	maximum

a Variables: TL, total lipid concentration; SL: LL ratio, solid: liquid lipid mass ratio.

### 2.2 Nanoparticle Characterization

#### 2.2.1 Dynamic Light Scattering and Nanotracking Analyses

The average diameters (size) and polydispersity indices (PDIs) of the nanoparticles were determined by dynamic light scattering (DLS) and zeta potentials (ZP) by electrophoretic mobility in a Nano ZS90 analyzer (Malvern Instruments, UK). The number of particles in suspension were measured by Nanotracking analysis (NTA) in an NS300 instrument (NanoSight Amesbury, UK), equipped with a green (λ = 532 nm) laser. For measurement, the samples were diluted in deionized water (*n* = 3).

#### 2.2.2 BZC Quantification, Determination of Encapsulation Efficiency, and Drug Loading

The quantification of BZC was carried out in a Varian ProStar high performance liquid chromatography (HPLC) equipped with a PS 325 UV–vis detector, a PS 210 solvent delivery module, and Galaxy Workstation software for data collection. The column was a Luna 5e C18 100 mm × 250 mm × 4.6 mm, with a flux of 1 mL/min. The mobile phase was composed of 70:30 methanol/water. The injection volume was 20 mL, and the absorbance was followed at 284 nm. The encapsulation efficiency (%EE) of benzocaine by the optimized NLC was determined by the ultrafiltration–centrifugation method (Müller et al., 2002), using 10 kDa molecular exclusion pore filters (Millex, Millipore). The amount of nonencapsulated BZC (BZC_free_) in the filtrates was quantified by HPLC, and the percentage of the encapsulated BZC was calculated according to eq 1
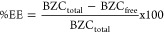
1

The amount of BZC encapsulated in the optimized nanoparticles was also expressed in terms of drug loading (%), according to eq 2(20,21)
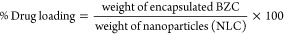
2

#### 2.2.3 Transmission Electron Microscopy Measurements

Transmission electron microscopy (TEM) and cryo-TEM were used to elucidate the morphology of the optimized NLC formulation (NLC_BZC_) and its control, prepared without benzocaine (NLC). For TEM, uranyl acetate (2%) was added to the diluted samples to provide contrast. After that, the aliquots were placed in copper grids coated with a carbon film and dried at room temperature. After drying, micrographs of the samples were analyzed at 60 kV in a Zeiss LEO 906 microscope (Zeiss, Freiburg, Germany). For cryo-TEM, a 300 Mesh Lacey carbon-supported copper grid (Ted Pella) was used. It was submitted to a light discharge of 15 mA for 10 s, using the Pelco easiGlow discharge system (Ted Pella, USA) to make it more hydrophilic. After that, the grids were put in a Vitrobot (Mark IV, Thermo Fisher Scientific, USA), where 3 μL of the samples was added and left for 20 s to be fixed. Then, an automatic transfer (2–3 s) was conducted to dry out sample’s excess, with a negative transfer force. The grid was plunged quickly into liquid ethanol and kept in a liquid nitrogen environment. The samples were analyzed in a JEOL-1400 Plus microscope, and images were treated with the Gatan Digital Suite (Gatan Inc., Pleasanton, California).

#### 2.2.4 Differential Scanning Calorimetry

Differential scanning calorimetry (DSC) thermograms of the optimized NLC formulation were obtained in a TA 2910 calorimeter and analyzed with Thermal Solutions v.125 software (TA Instruments, Delaware). The samples were dried and heated at a rate of 10 °C min^–1^ from 30 to 200 °C.^22,23^ Optimized nanoparticles (NLC_BZC_) and their control without benzocaine (NLC), besides their main ingredients (CP, P68, and BZC), were run.

#### 2.2.5 X-ray Diffraction

Powder X-ray diffraction (XRD) data were obtained from a Shimadzu XRD7000 diffractometer, using a Cu Kα source, operating at 1.5418 Å, with a scan step of 1° min^–1^, between 2θ values from 5 to 50°. Samples of dried NLC_BZC_, NLC, physical mixture, CP, P68, and BZC were analyzed at ambient temperature.

#### 2.2.6 Small-Angle Neutron Scattering

Neutron scattering measurements (SANS) were performed at a V16 time-of-flight (very small-angle scattering) instrument in the Helmholtz Zentrum at Berlin, Germany, in two configurations related to the distance from the detector to the sample: 1.7 m with neutron wavelengths of 1.8–3.8 Å and 11 m with neutron wavelengths of 1.6–9.2 Å. The samples were placed in Hellma 110 QS cuvettes, and a waiting time of 30 min between acquisitions was adopted to ensure temperature stabilization. NLC_BZC_ and NLC samples were prepared in D_2_O to reach a significant contrast between the solvent and the nanoparticles, and measured at 25, 37, and 40 °C. Corrections for sample transmission, background detector counts, empty cell scattering, and detector efficiency were included in the final data reduction. SANS data were radially averaged and merged to give a total *q* range of 0.005–0.5 Å^–1^.^24^

To model the SANS data to get a further understanding of the optimized nanoparticles, the empirical function in eq 3 was applied
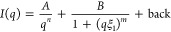
3where *A* and *B* are constants, *n* and *m* are the power-law indices, back is the incoherent background, and ξ_1_ is the wave correlation length.^25−27^ The first term in the equation is a power-law decay, known as Porod-like scattering, that describes the scattering from clusters or aggregates (aggregate contribution) in the system. The second term is the Lorentzian function that corresponds to the scattering from the hydrophobic interactions between the lipids that form the NLC (association contribution), where the key parameter is ξ_1_.

#### 2.2.7 Raman Imaging

The miscibility of excipients with BZC was evaluated by Raman imaging in a preformulation sample. All compounds were heated to 64 °C. 0.3 g of BZC was mixed with 1.0 g of propylene glycol monocaprylate (LL) until homogenization. This mixture plus 0.5 g of P68 were added into 1.5 g of melted cetyl palmitate (SL) and manually agitated until they formed a homogeneous solution. This sample was deposited in an aluminum Petri dish, and the Raman data were acquired after cooling to room temperature (25 °C) for 30 min.

Raman images were collected in a Via Qontor confocal Raman microscope (Renishaw, Gloucestershire, UK). The exposition time was 5.0 s, 1 exposition/pixel, 5× objective, a laser excitation of 785 nm, and a laser power of 10 mW, in a spectral range of 715–1806 cm^–1^, at a 1 cm^–1^ spectral resolution and a step size of 20 μm, on both *x* and *y* axes. The Raman maps were obtained in the same area but at three different temperatures: 27.0, 37.0, and 40.0 °C using the THMS600 (Linkam Scientific, UK) temperature controller at a rate of 10 °C min^–1^.

Cosmic rays were removed from Raman spectra using an appropriate algorithm.^28^ The baseline was corrected using asymmetric least-squares. The Savitzky–Golay algorithm (5 points window and second-degree polynomial) was employed to reduce the noise, and the data set was normalized using a unit vector. The chemical maps were built using classical least-squares (CLS), a very useful bilinear model for chemical image treatment. In this algorithm, a mixture spectrum was considered as a linear combination of pure compound spectra.^29^ The data cubes were unfolded into a matrix, and the excipients and BZC spectra were used as input *S*. Afterward, the scores matrix was refolded and generated chemical maps representing the distribution of compounds on the analyzed surface. For data preprocessing and CLS analysis, MATLAB version 8.3 (MathWorks Inc., Natick, Massachusetts) and PLS toolbox version 8.6.2 were used.

### 2.3 Shelf-Stability Studies

The physicochemical stability of NLC formulations was followed for 12 months at room temperature (25 ± 2 °C) and 60 ± 5% humidity. The analyzed parameters were nanoparticle size (nm), PDI, ZP (mV), number of particles/mL, pH, and %EE. Analysis of variance (ANOVA, 95% confidence level) and Tukey post hoc test were used to compare significant differences, regarding the initial time and subsequent measurements.

### 2.4 In Vitro Release Tests

The release of BZC from the nanoparticles was measured in a Franz diffusion cell system composed of donor (400 μL) and acceptor (15 mL) compartments, separated by a 0.1 μm pore size polycarbonate membrane (Nucleopore Track-Etch, Whatman). The acceptor compartment was filled with a mixture of propylene glycol/deionized water (70:30 v/v). The release of benzocaine encapsulated in the optimized NLC (NLC_BZC_) was compared to that of a solution of BZC dissolved in the same mixture. The system was kept at 37 °C under magnetic stirring (400 rpm). At predetermined intervals for 25 h, aliquots (200 μL) were taken from the acceptor compartment for HPLC analysis, and the volume was immediately replaced with buffer solution to keep the sink condition. All measurements were performed in triplicate, and the amount of BZC in the acceptor compartment was quantified by HPLC (see Section 2.2.2).

The KinetDS 3.0 software was used for the quantitative analysis of the obtained release curves (Mendyk et al., 2012). Among the several kinetic models tested (zero order, first order, Higuchi, Korsmeyer–Peppas, Hixson Crowell, Weibull, and Baker–Lonsdale) and according to the determined *R*^2^ coefficient, the best fit was observed with the Baker–Lonsdale model, described by eq 4(30,31)
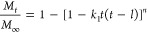
4where *M_t_* and *M*_∞_ refer to the quantity of released BZC in time “*t*” and infinity time, respectively.

### 2.5 In Vivo Anesthetic Effect

Adult male Swiss mice (30–35 g) obtained from CEMIB-UNICAMP (Centro de Bioterismo da Unicamp, Brazil) were used. The animals (4 animals per cage) were submitted to 12 h light/dark cycles, with water and food ad libitum at 22 ± 3 °C, for 7 days prior to tests. The animals were randomly separated (*n* = 7) to receive the treatment. The antinociceptive effects of the formulations were determined by the tail-flick test, following a protocol approved by the Unicamp Institutional Animal Care and Use Committee (#5428-1/2019) that obeys the IASP rules.

The samples were topically administered at the tails’ base in the region of the caudal nerve. To turn the formulations suitable for topical use, a 5% Carbopol gel was prepared and the samples were dispersed into the gel prior to polymerization. The tested samples were 1.5 and 3% NLC_BZC_ gel, 3% free BZC in gel, 20% Benzotop (commercial BZC), and Carbopol gel (blank).

Tail-flick test*:* for baseline determination, the animal was placed in a horizontal restraint support and a portion of the tail (2 cm from the tip) was exposed to an analgesimeter with a projector lamp (55 ± 1 °C) connected to a control switch and a timer. The time interval between temperature exposure and tail-flick was recorded (in seconds) and referred to as the latency time. A cutoff time of 10 s was used to prevent any thermal injury to the tail tissue. Baseline latency times were recorded for all animals prior to the experimental treatment. Results were expressed as the percentage of maximum possible effect (% MPE), duration of the analgesic effect (min), and area under the efficacy curve for each experimental group.^32,33^ The area under the curve (AUC_0–25_) of the analgesic effect was calculated through the % MPE plot. Statistical analyses were performed by one-way ANOVA with the Tukey–Kramer post-test, using GraphPad Prism software version 8.00 for Windows (La Jolla, California).

## 3 Results and Discussion

The selection of a suitable composition (lipid matrix plus surfactant) is the first step in the development of nanostructured lipid carriers. Several types of lipid mixtures have been described in the literature for NLC preparation, which are compatible with biomedical applications.^14,16,34−36^ The main criteria for the choice of NLC excipients is the solubility of the drug in the lipid matrix,^37^ but formulations with appropriate physicochemical features such as low particle size and polydispersity and ZP different from zero were also important.^38,39^ In the case of BZC, preliminary tests were conducted with 3 kinds of solid and liquid lipids, as shown in Table S1, Supporting Information, and the best fit for such criteria was found with cetyl palmitate (SL) and propylene glycol monocaprylate (LL), the proportion of which was established through DoE. The total lipid concentration (20%) and the tested SL/LL lipid ratios (60:40–80:20) took into account the previous experience from the group, as well as the choice of P68 (5%) as a surfactant.

### 3.1 Design of Experiments

**Figure 1:**
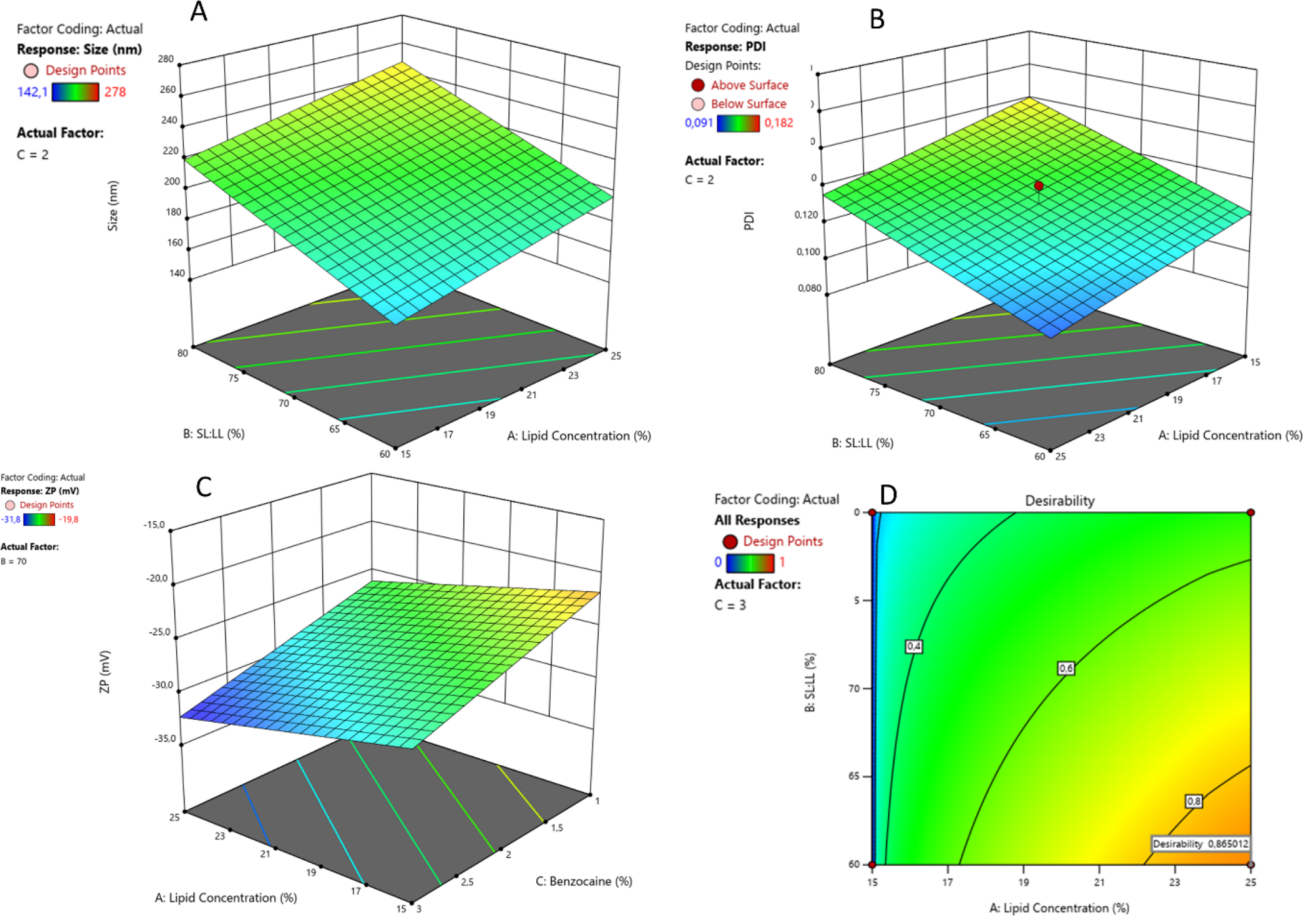
Surface graphs for the studied responses as a function of the significant variables: (A) particle size, (B) PDI, and (C) ζ potential. (D) Desirability graph for the NLC containing benzocaine.

DoE is a multivariate tool useful in pharmaceutical development, particularly in the optimization of formulations. By employing this tool, it is possible to efficiently test various combinations of excipients and proportions, saving time and resources. This is especially significant in the field of nanotechnology, where the interactions between excipients and drugs can significantly impact key parameters such as nanoparticle size, polydispersity index, and ζ potential.^40^ A 2^3^ factorial design was performed in triplicate at center point (11 formulations in total) for the NLC plus BZC system. The Design Expert software was used to perform the design, with variables and responses as given in Table 1. For optimizing the formulation designed for the topical application, the chosen criteria included particles of smaller sizes (for better skin permeation), minimal PDI (monodisperse systems), and higher ZP values—in module.^38,39^ As evaluated by ANOVA, significant linear mathematical models were obtained without lack of fit these models described the influence of the excipients and their interactions on the properties of interest (Table 2) and in the response-surface graphs in Figure 1A–C.

**Table 2:** Significant Positive and Negative Effects on the Properties of Interest in the 2^3^-Experimental Design of Benzocaine-Loaded NLC^1^

response	range of values	positive effect	negative effect
size (nm)	142.1–278.0	A, B, C	AC
polydispersity index	0.080–0.182	B and BC	A
ζ potential (mV)	–19.8 to −31.8	AC	

1 Variables: A = TL; B = SL/LL ratio; C = BZC.

The particle sizes (140–280 nm) of the NLC lay in a suitable range for infiltrative DDS administration, and all tested variables had a positive effect on it (the increase of their levels caused an increase on the response). As expected, the greater the concentration of the major NLC components (TL) is, the greater the nanoparticle size. Also, the increase in the SL/LL ratio had a positive effect. Therefore, a high concentration of cetyl palmitate (SL) possibly reflected in higher lipid viscosity, reducing the efficacy of the homogenization process, resulting in particles of higher size.^41−45^

The polydispersity index (PDI) gives an indication of the size homogeneity in the system, with values in the range of 0.1–0.2 reflecting a monodisperse distribution,^39^ as observed here. The SL:LL ratio and its interaction with BZC had positive effects on PDI (Table 2), whereas the total lipid showed a negative effect (decreased polydispersity).

The ζ potential is a parameter inferred from the electrophoretic mobility of nanoparticles that is related to the surface charges at the Stern–Volmer layer. ZP values give an indication of the repulsion forces between nanoparticles, and the higher it is (in modulus), the lower the aggregation tendency. The determined ZP lied between |19.8 and 31.8| mV, what is compatible with good shelf stability.^42,46^ Since BZC interacts differently with the solid and liquid lipids in the NLC core (see XRD, SANS, and Raman imaging data), we hypothesize that the more negative ZP values observed at higher lipid and BZC concentrations reflect the lipid displacement promoted by benzocaine. This results in a more superficial distribution of cetyl palmitate at the slipping plane of the NLC, as previously observed with the BZC analogue butamben.^47^

Based on the results for each response, the desirability region shown in Figure 1D was determined. The optimal formulation was composed of 25% TL, 60:40 SL/LL ratio, and 3% BZC plus 5% P68, and subsequent experiments were conducted with this selected composition.

The optimized NLC_BZC_ formulation showed particles with 180.8 ± 3.6 nm average diameters, 0.090 ± 0.024 polydispersity indices, and negative |35.2 ± 0.8 mV| ζ potentials (Table S2). The size and homogeneity of the size distribution (Span index < 0.8) of the nanoparticles were confirmed by NTA, which also revealed the number of particles in suspension: 1.9 × 10^14^/mL (Table S3). Interestingly, both technique (DLS and NTA) data revealed a slight increase in the nanoparticle average size after drug encapsulation (NLC vs NLC_BZC_).

### 3.2 Encapsulation Efficiency and Drug Loading Capacity of the NLC

Encapsulation efficiency (%EE) describes the nanocarriers’ ability to encapsulate the drug. The lipid matrix of the optimized NLC showed excellent capacity to encapsulate BZC: 96.0 ± 0.4%, corresponding to a drug loading capacity of 11.52% (eq 2). Similar results (loading capacity = 11%) were reported for the ester-type-LA tetracaine^35^ encapsulated in NLC. The high upload capacity of the NLC is probably due to the hydrophobic character of BZC and its compatibility with the chosen mixture of solid and liquid lipids, whose low crystallinity index afforded spaces to accommodate BZC molecules^48,49^ in the NLC core.

These results confirm the efficiency of the NLC optimization process and the high lipophilicity of BZC, which was inserted within the lipid matrix of the optimized nanoparticles.

### 3.3 TEM and Cryo-TEM Analyses

**Figure 2:**
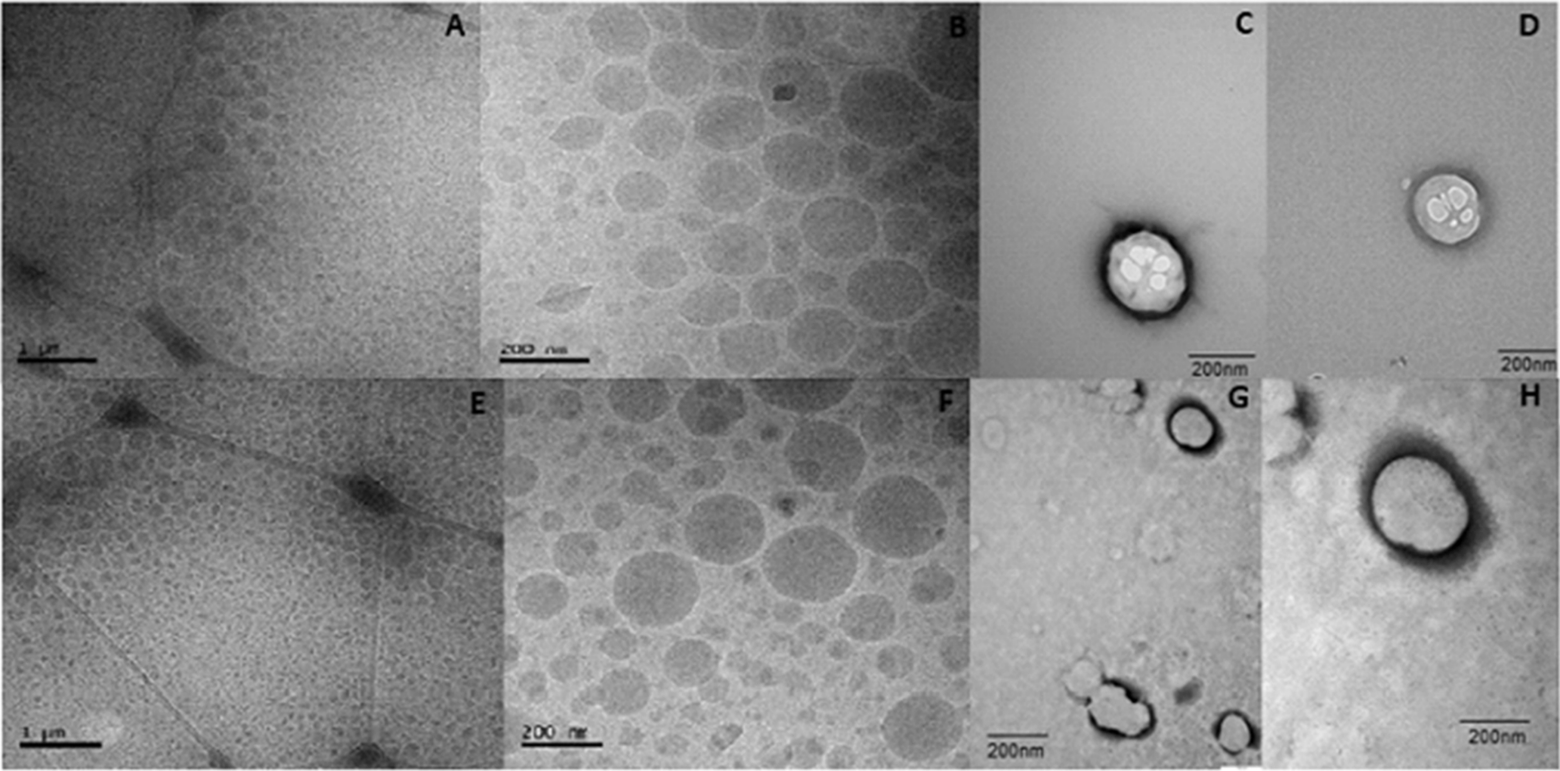
Cryo-TEM and TEM images of the optimized NLC formulations: without (A–D) and with BZC (E–H). Cryo-TEM images (A, B, E, and F). Scale = 1 μm (A, E) and 200 nm (B, F). TEM images (C, D, G, and H) at magnitudes of 60.000× (C, G) and 100.000× (D, H).

Information about the morphology of NLC was obtained by TEM and cryo-TEM. Both techniques (Figure 2) revealed spherical nanoparticles with regular and defined borders. Addition of BZC did not change the NLC morphology. Moreover, the observed sizes of the nanoparticles agreed well with those determined by DLS (Table S2 and Figure 1A) and NTA (Table S3).

### 3.4 Calorimetric, X-ray Diffraction, Small-Angle Neutron Scattering, and Raman Analyses

To further analyze NLC and to correlate its internal excipients’ arrangement with drug encapsulation, DSC, XRD, SANS, and Raman imaging analyses were conducted. Differential scanning calorimetry was performed for BZC, NLC major excipients (CP, P68), their physical mixture, and the optimized NLC formulation, with and without BZC (Figure S1, Supporting Information).

The thermograms of BZC, CP, and P68 revealed sharp endothermic peaks at 92.3, 56.4, and 54.7 °C, respectively, related to their melting points.^15,50^ For NLC, just one thermal event was observed at 53.0 °C, probably reflecting the melting transition of CP, the major component of the nanoparticle. When BZC was added, there was a slight decrease in this transition temperature (from 53.0 °C in NLC to 52.9 °C—NLC_BZC_) and enthalpy (from 190.8 to 164.7 J/g). These data also show that the fraction of BZC encapsulated in the NLC (96%, according to %EE) decreased the crystallinity of the lipid core (NLC_BZC_ vs NLC), as observed previously, with other local anesthetics encapsulated in NLC.^51−54^ The absence of the melting point of pure BZC in the NLC_BZC_ sample is another indicator of BZC dissolution into the lipid matrix of the NLC.

**Figure 3:**
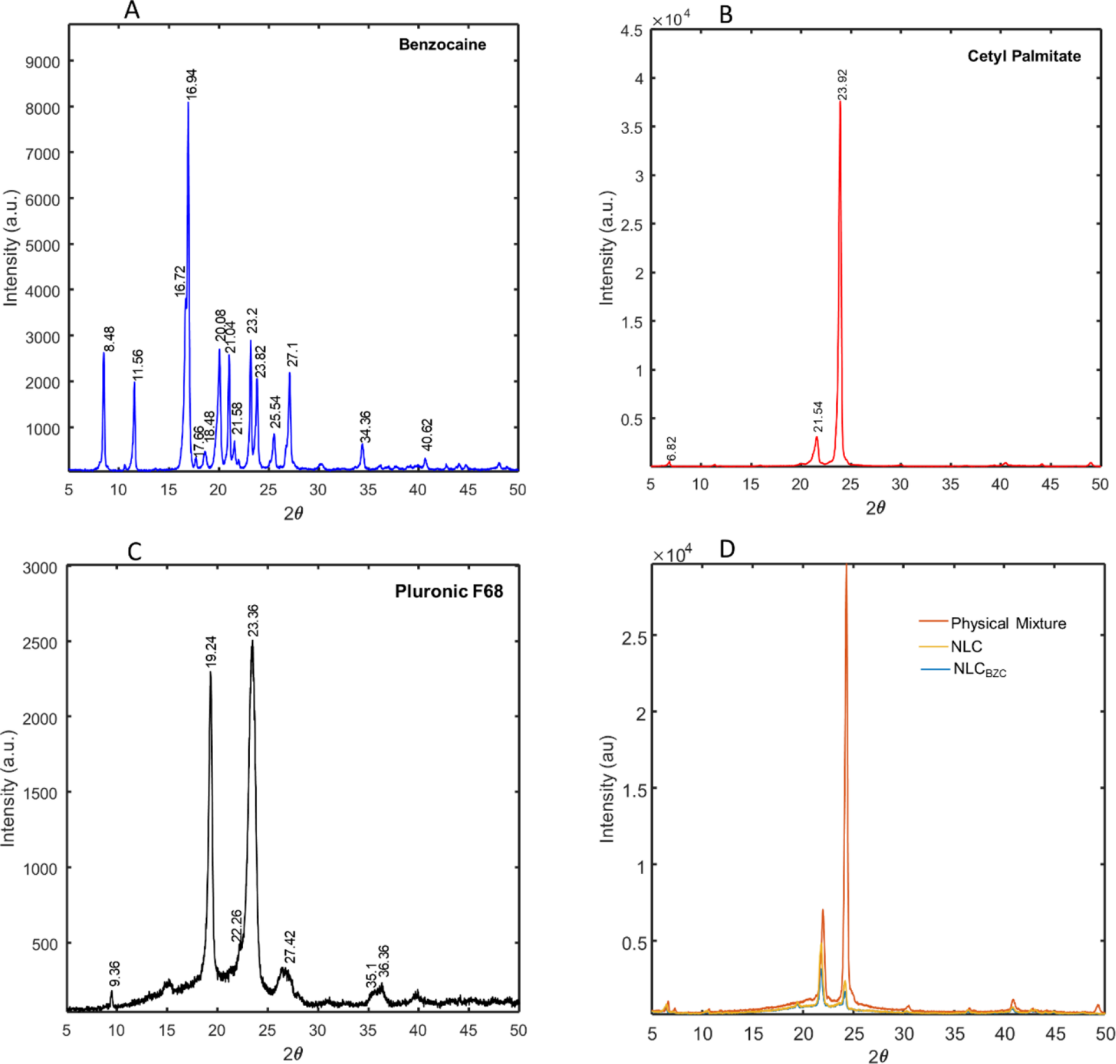
X-ray diffractograms for the samples: BZC (A), CP (B), P68 (C), physical mixture, and NLC and NLC_BZC_ (D). Data were obtained at an ambient temperature, with a Cu Kα source, at a scan step of 2° min^–1^.

Powder X-ray diffraction (XRD) is a useful technique to reveal polymorphic structural changes in NLC dispersions.^35^ X-ray measurements were carried out for BZC, NLC major excipients (CP and P68), physical mixture, and the optimized NLC, with and without BZC (Figure 3). The diffractogram of BZC confirmed its crystalline nature.^55,56^ The XRD of the major lipid excipient CP showed intense reflexions at 21.54 and 23.92°, which indicates its crystalline structure.^11^ No replections from BZC were noticeable in the optimized NLC formulation sample (Figure 3D), and there was a loss in intensity (from the physical mixture to NLC_BZC_) compatible with the insertion of BZC into the lipid matrix.^54^

**Figure 4:**
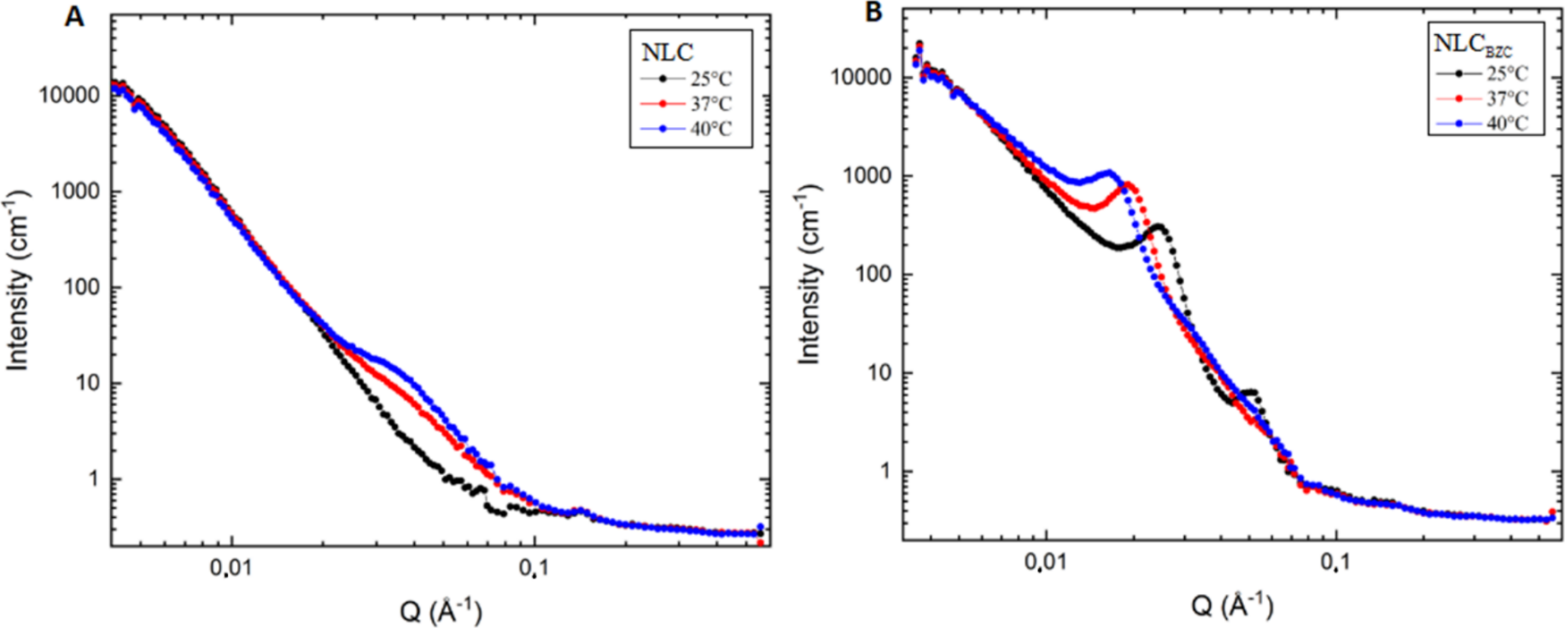
SANS data measured at 25, 37, and 40 °C for (A) control NLC and (B) optimized (NLC_BZC_) formulation.

Small-angle neutron scattering has become a routine technique for the structural characterization of macromolecular assemblies of polymers, micelles, and even NLC^35^ in the spatial range of a few to hundreds of nanometers. SANS measurements were performed to probe the structural organization of the optimized NLC, with and without benzocaine (Figure 4).

SANS data, collected at 25, 37, and 40 °C, revealed several systematic tendencies in the internal arrangement of the NLC. The behavior of NLC without BZC (Figure 4A) was modeled using the Guinier–Porod equation, while that of NLC_BZC_ (Figure 4B) used eq 3 (see Section 2). BZC inclusion into the nanoparticles significantly affected the packing of the lipids. A peak in the 0.1396 Å^–1^ position was observed in the NLC_BZC_ sample but not in the control (NLC). Such a peak, related to a 45.0 Å net parameter, denotes the existence of a lamellar arrangement in the lipid matrix, induced by BZC. Lamellar arrangements in the lipid core of NLC prepared with cetyl palmitate (as in here) have also been observed after incorporation of the local anesthetics dibucaine,^53^ tetracaine,^35^ or the antineoplastic docetaxel^57^ into the nanoparticles. Moreover, the half-height width of this peak diminished from 0.11 (25 °C) to 0.03 Å^–1^ (40 °C), indicating an increase in the crystallite size.

Interestingly, the Guinier–Porod exponent, providing information on the nanoparticle surface, remained the same: −4.0 (NLC) and −4.2 (NLC_BZC_) at 25, 37, and 40 °C. According to it, both NLC and NLC_BZC_ exhibited smooth surfaces, which can indicate drug dispersion into the lipid matrix, without the adsorption of unencapsulated molecules in the nanocarrier surface.^58^ BZC did not change the radius of gyration nor change the surface of the nanoparticle, as observed in other NLC systems,^57^ confirming the dispersion of the anesthetic inside the nanoparticles.

The lamellar structures in the interior of the NLC agree with DSC and XRD data that revealed a decrease in the crystallinity of the lipid core (Figure 3) and give clear evidence that BZC interacts differently with the solid and liquid lipids. Indeed, in a previous Raman imaging study with NLC composed of CP and propylene glycol monocaprylate, the interaction of butamben (*n*-butyl-*p*-aminobenzoate) with the liquid lipid (but not CP) was demonstrated.^47^

**Figure 5:**
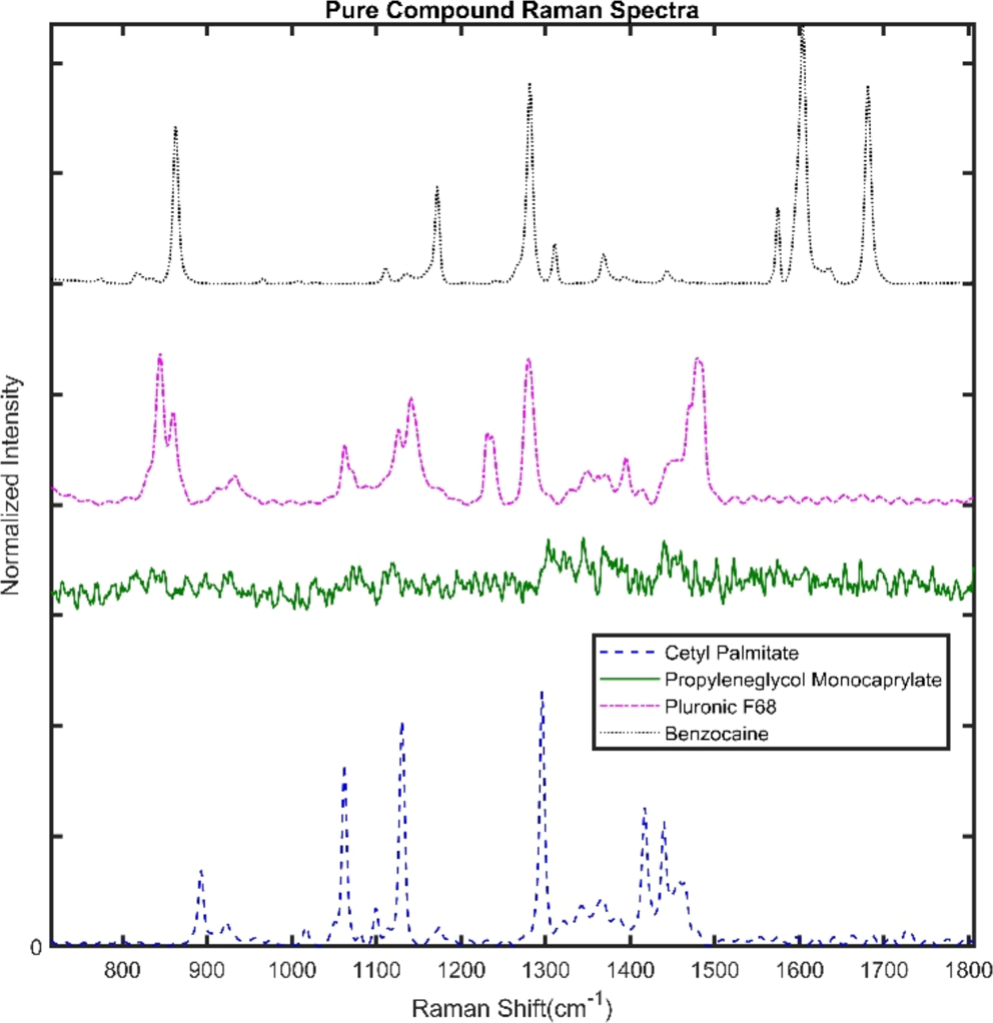
Raman spectra of cetyl palmitate, propylene glycol monocaprylate, P68, and BZC.

Raman analyses were used to evaluate the miscibility of BZC with the NLC excipients in a preformulation.^59^Figure 5 shows the Raman spectrum of BZC and each NLC excipient. The spectrum of propylene glycol monocaprylate (green) was very noise—because of the lower LL proportion in the formulation—but it shows bands around 1300–1500 cm^–1^, related with −CH_2_ bonds^60^ and COOH-carbonyl groups.^61^ The spectra of the solid compounds (CP, P68, and BZC) displayed sharper and thinner vibrations than those of the LL because of higher order. The BZC signal at 1680 cm^–1^ is assigned to its carbonyl group, while signals at 1600 and 1573 cm^–1^ are related to the C=C bonds and those at 1630 and 1277 cm^–1^ to N–H and C–N bonds, respectively.^62^

**Figure 6:**
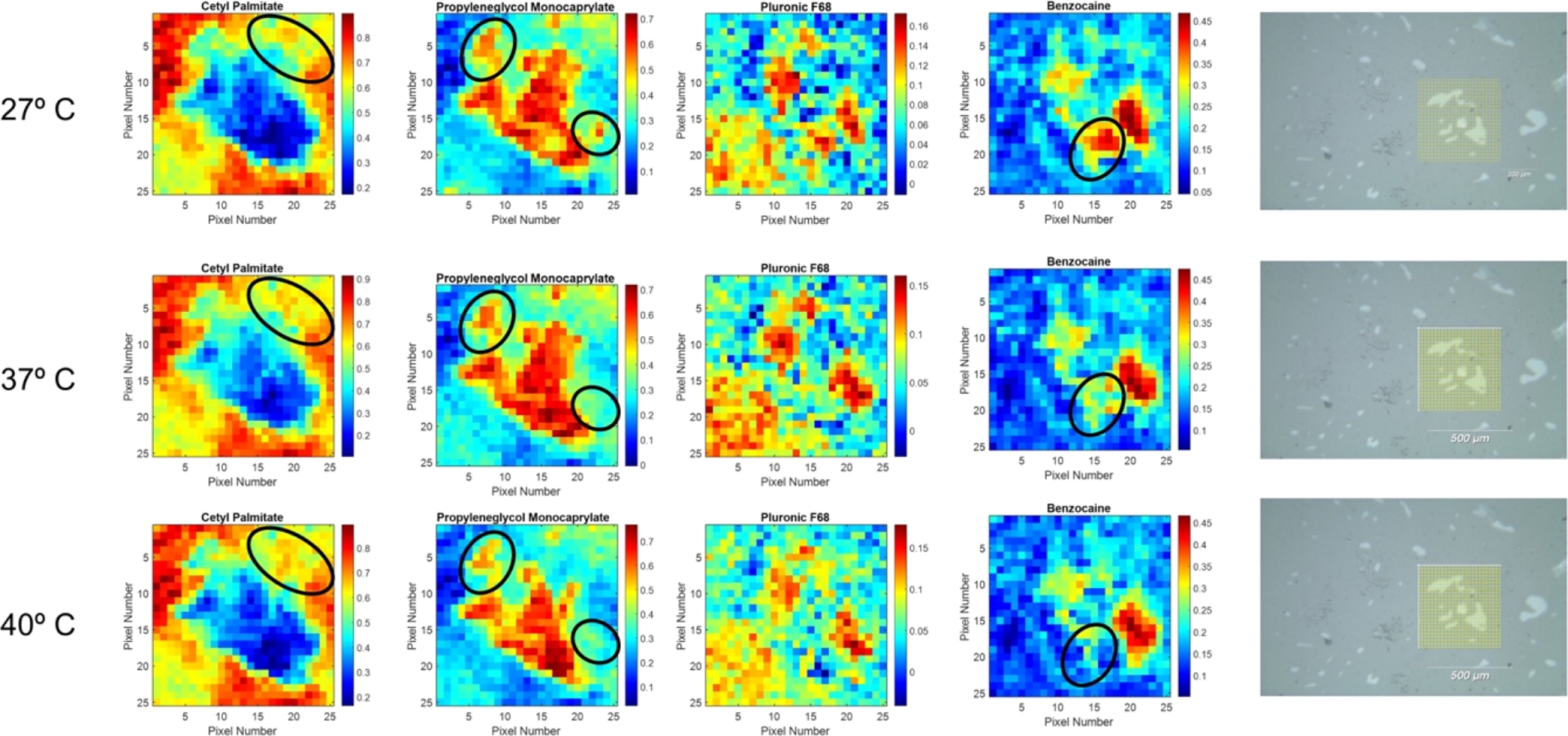
Chemical maps obtained at 27.0, 37.0, and 40.0 °C for the NLC components (cetyl palmitate, propylene glycol monocaprylate, P68) and BZC plus visible images (right), where the yellow square represents the sampled area. The black ellipses highlight areas of maximum changes according to the temperature (see the text).

The chemical maps obtained after CLS analyses are shown in Figure 6, as well as the Raman images, at 27.0, 37.0, and 40.0 °C. According to the bar, colors near red (blue) represent regions with higher (lower) concentrations. It is possible to see that cetyl palmitate (SL) is mainly in the border area, while the LL and BZC are mostly in the central part of the sample, and P68 can be found in both regions. Looking at the visible images (Figure 6, right), the gray part of the sample is composed mainly of CP and P68; the white part is where the LL and BZC are concentrated, but some P68 can be found, as well. The white part of the sample melted while increasing the temperature. As for the chemical maps, the blue spots on CP maps seemed to have melted and decreased at increasing the temperature from 27.0 to 40.0 °C (as highlighted by black ellipses in Figure 6). Similarly, the red areas on the LL and BZC maps also decreased at higher temperatures, while P68 showed a more homogeneous distribution. SANS data (Figure 4) have pointed to the formation of lamellar structures inside the NLC, with increased interlamellar distances at higher temperatures. The Raman imaging data in Figure 6 corroborate the changes in Q values observed by SANS, showing that the incorporation of P68 and BZC between the lipids is favored at higher temperatures.

### 3.5 Shelf-Stability Analyses

**Figure 7:**
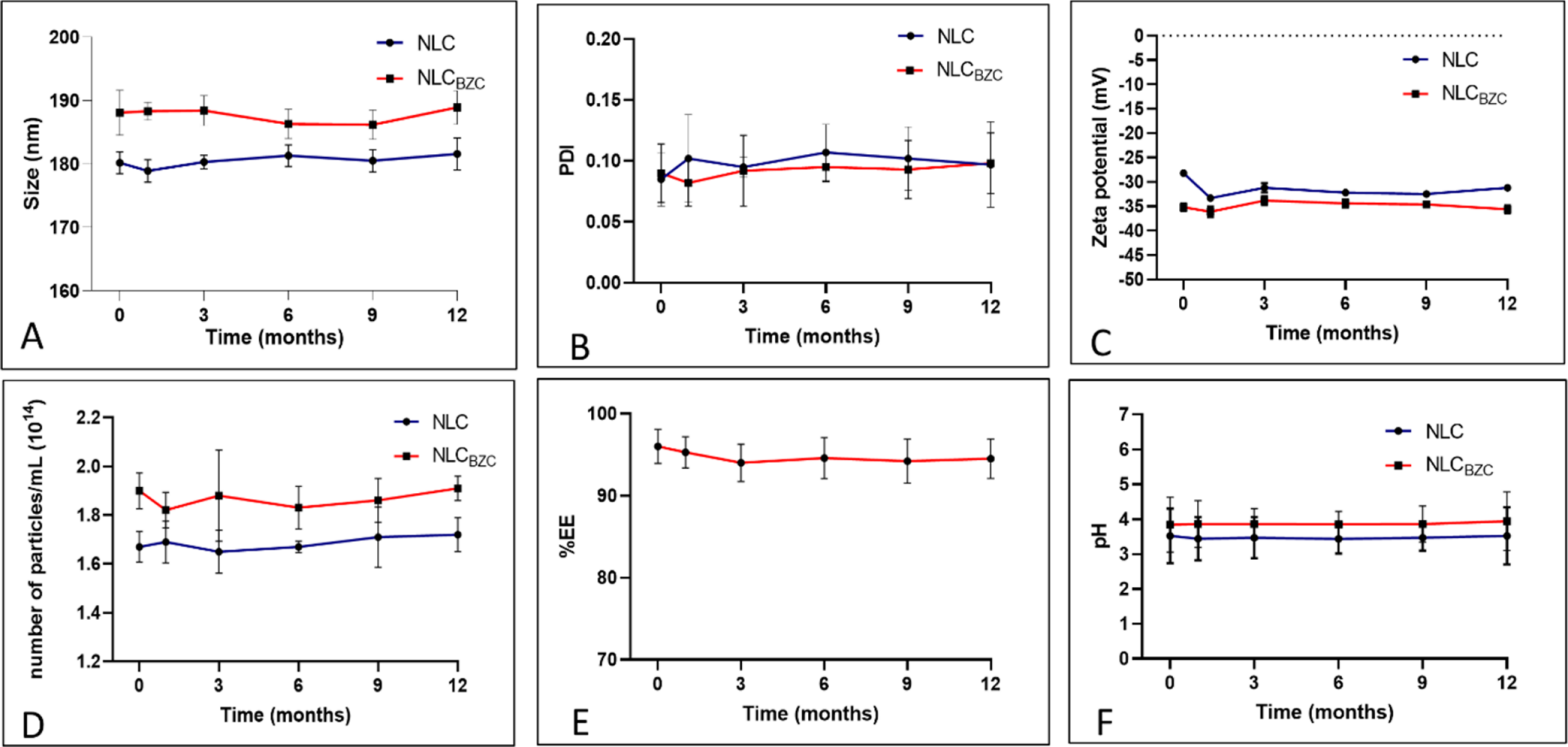
Physicochemical stability of the optimized formulation (NLC_BZC_) and its control (NLC) over time and at room temperature. (A) Size, (B) PDI, (C) ζ potential, (D) number of particles/mL, (E) %EE, and (F) pH. One-way ANOVA: *p* < 0.05.

Physicochemical evaluation of pharmaceutical nanoformulations is an essential step to confirm that the structural properties were preserved over time. Therefore, the optimized formulation and its control (without BZC) were stored at room temperature (25–30 °C) and observed for 12 months, regarding size, PDI, ZP, number of particles/mL, %EE, and pH. Even after 1 year of storage, no statistically significant differences were observed for any parameter (Figure 7), in comparison to freshly prepared samples. Moreover, the encapsulation of BZC did not modify the colloidal system (NLC_BZC_ vs NLC), keeping it stable during storage.

### 3.6 In Vitro BZC Release Curves

**Figure 8:**
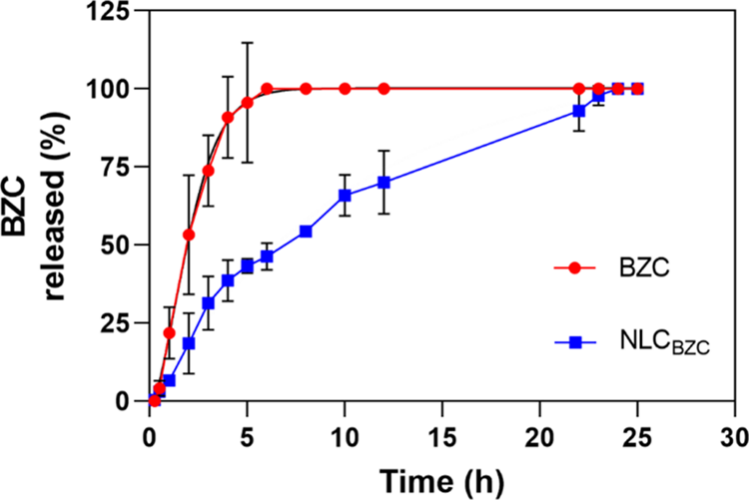
In vitro release profile of benzocaine: free (BZC) or encapsulated (NLC_BZC_), measured at 37 °C.

In vitro release kinetics tests shed light on the drug release profile of the DDS. Using vertical diffusion Franz cells, the release of BZC was followed for a period of 25 h at 37 °C (Figure 8).

Free BZC was completely released after 6 h of experiment, in agreement with previous reports in the literature.^63,64^ As for the kinetics of BZC released from the optimized formulation, it reached 70% of release after 12 h of experiment and 100% in 24 h. So, as also observed for other LA agents (dibucaine,^53^ tetracaine^35^), the optimized NLC formulation significantly reduced BZC release, compared to its control.

The release curves were analyzed with Kinect DS3 software. Using different mathematical models (Table S4) and accordingly to the coefficient of determination (*R*^2^ = 0.9472), the Baker–Lonsdale equation offered the best fit, regarding the release of benzocaine from the NLC_BZC_. This result is reasonable since the Baker–Lonsdale model, a derivation of the Higuchi model, describes a sustained drug release from a spherical matrix, combining diffusion and nanoparticle degradation as the responsible factors for the sustained release mechanism.^31,65^

### 3.7 In Vivo Anesthetic (Tail-Flick Test) Effect of NLC_BZC_

**Figure 9:**
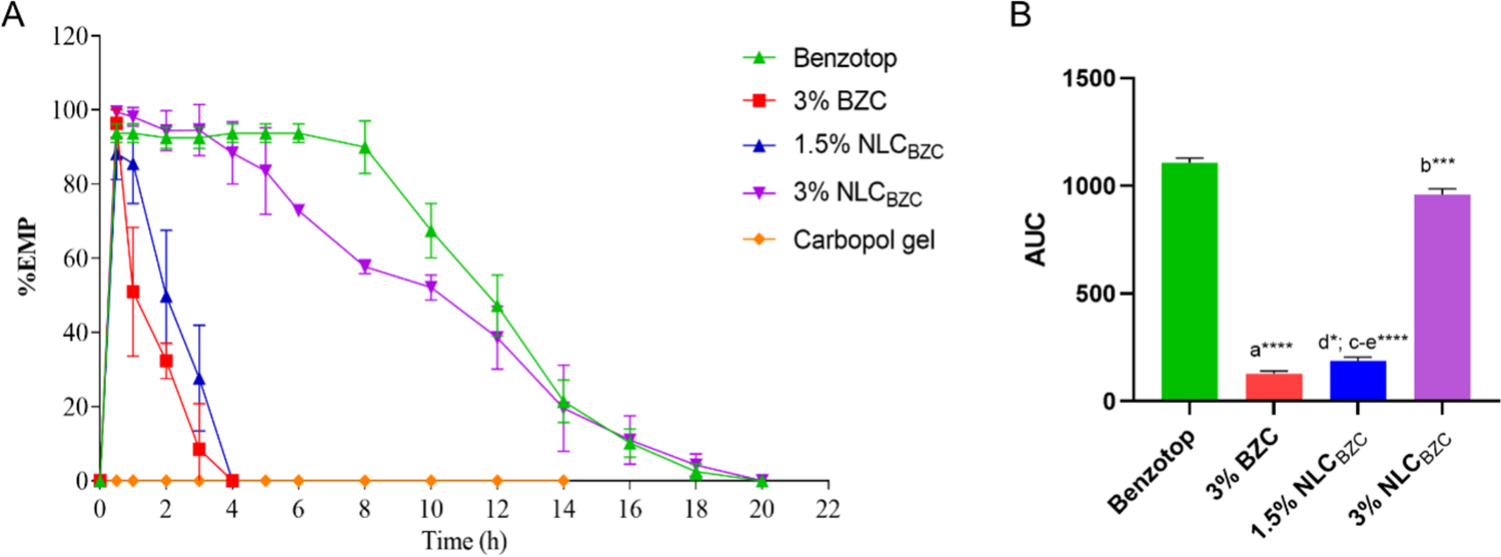
Tail-flick antinociceptive test. (A) Maximum possible effect of BZC (free and encapsulated in NLC) gel topically applied to the tail-base of adult Swiss mice, in comparison to the effect promoted by the commercial Benzotop formulation. Statistic analysis by one-way ANOVA plus Tukey–Kramer post hoc, *p* < 0.05. (B) Area under the (time-effect) curve after topical gel application of Benzotop (20% benzocaine), 3% free benzocaine (BZC), and optimized formulations: 1.5 and 3.0% NLC_BZC_. Statistical analysis: one-way ANOVA with the Tukey–Kramer post hoc: (a) 3% BZC × Benzotop; (b) Benzotop × 3.0% NLC_BZC_; (c) 3.0% NLC_BZC_ × 1.5% NLC_BZC_; (d) 3%BZC × 1.5% NLC_BZC_; (e) Benzotop × 1.5% NLC_BZC_. *p < 0.05, ***p < 0.001, and ****p < 0.0001.

The tail-flick test, described by D’Amour and Smith,^66^ was used to evaluate the antinociceptive activity of the samples, which were topically applied to the tail-base region of male adult Swiss mice. No skin irritation was observed in the animals treated with the formulation or its control. The maximum antinociceptive effect (% EMP) achieved after the administration of 3% free benzocaine or NLC_BZC_ (at 1.5 and 3.0%) incorporated in 5% Carbopol gel is shown in Figure 9A, in comparison to that elicited by Benzotop, a commercial BZC gel formulation.

As expected, the 5% Carbopol gel (blank) produced no anesthetic effect. All of the other tested gels induced anesthesia after 30 min of application. The maximum effect observed with free BZC (3.0%) and 1.5% BZC encapsulated in NLC_BZC_ occurred in 1 h, and the return to basal values was after 4 h. In a different manner, 3% NLC_BZC_ showed an anesthetic effect that lasted for 18 h, equivalent to the effect of Benzotop, the commercial product that contains 20% BZC. These exciting results confirm the increase in BZC bioavailability achieved with vehiculation in the lipid nanoparticles. Indeed, 3% NLC_BZC_ evoked topical anesthesia similar to that of Benzotop, with ca. 7 times higher LA concentration, as also shown in the AUC plots of Figure 9B. The potentiation of the anesthetic effect after encapsulation in NLC has also been observed with other LA agents such as lidocaine, prilocaine, tetracaine, butamben, articaine, and bupivacaine.^10,12,14,15^ In the case of BZC, the risk of systemic toxicity associated to PABA formation is significantly reduced with the use of lower (3%) benzocaine concentrations. Finally, 3% is the clinical concentrations for infiltrative anesthesia of lidocaine, prilocaine, mepivacaine, and articaine^67^ so that the 3% NLC_BZC_ formulation opens the possibility of using benzocaine by the infiltrative route.

## 4 Conclusions

An innovative lipid-based DDS designed for the delivery of benzocaine was developed through the utilization of DoE. The optimized formulation, characterized by different biophysical techniques, exhibited favorable physicochemical properties, prolonged shelf stability and enhanced the bioavailability of BZC, a LA exclusively administered via the topical route. NLC_BZC_ effectively promoted a sustained drug release (ca. 20 h), and a prolonged anesthetic effect of benzocaine (∼18 h), in mice. In this way, NLC_BZC_ reduced the required effective BZC concentration for topical anesthesia, thereby diminishing side effects such as methemoglobinemia and allergic reactions associated with benzocaine use. While further research and development in this area are warranted to fully explore the potential of NLC as a DDS for local anesthetics, the proposed lipid-based nanoformulation opens new possibilities for the administration of benzocaine through the infiltrative route.

Finally, the results here show how different scattering techniques can afford complementary information and be used together, providing details on the physical, chemical, and structural changes in the organization of DDS, such as the preferential binding of BZC with the liquid lipid, propylene glycol monocaprylate.

## Supporting Information Available

DSC thermograms (Figure S1); preliminary tests for the preparation of NLC_BZC_ employing different solid and liquid lipids (Table S1); DLS: average particle size, polydispersity index, and zeta potential (Table S2); NTA: average and cumulative data of particle size (Table S3); and mathematical models applied to the release kinetic curves of NLC and NLC_BZC_ (Table S4) (PDF)
