# Supplementary material for: Fresh Carrier for an Old Topical Local Anesthetic: Benzocaine in Nanostructured Lipid Carriers
Source: ACS Biomater Sci Eng. 2024 Jul 29;10(8):4958–69. doi: 10.1021/acsbiomaterials.4c00585 (PMC11322916; doi:10.1021/acsbiomaterials.4c00585)
Supplement: Supplementary file 1 — ab4c00585_si_001.pdf [file ab4c00585_si_001.pdf]

SUPPLEMENTARY MATERIAL

FRESH CARRIER FOR AN OLD TOPICAL LOCAL ANESTHETIC: BENZOCAINE IN NANOSTRUCTURED LIPID CARRIERS

Souza, A.D.[1], Rodrigues da Silva, G.H.[1,2], Ribeiro, L.N.M.[1], Mitsutake, H.[1,3], Bordallo, H.N.[3], Breitkreitz, M.C.[4], Lima Fernandes, P.C.[1], Moura, L.D.[1], Yokaichiya, F.[5], Franco, M.[6] de Paula E.[1,*]

[1] Departamento de Bioquímica e Biologia Tecidual, Instituto de Biologia, Universidade Estadual de Campinas (Unicamp), ZIP 13083-862, Campinas, SP, Brazil.

[2] Laboratório Nacional de Biociências, Centro Nacional de Pesquisa em Energia e Materiais, ZIP 13083-100, Campinas-SP, Brazil.

[3] Niels Bohr Institute, University of Copenhagen, ZIP 2100 Copenhagen, Denmark.

[4] Departamento de Química Analítica, Instituto de Química, Unicamp, ZIP 13083-862, Campinas, SP, Brazil.

[5] Departamento de Física, Universidade Federal do Paraná (UFPR), ZIP 81531-980, Curitiba, PR, Brazil.

[6] Instituto de Pesquisas Energéticas e Nucleares, IPEN-CNEN/SP, ZIP 05508-000, São Paulo, SP, Brazil.

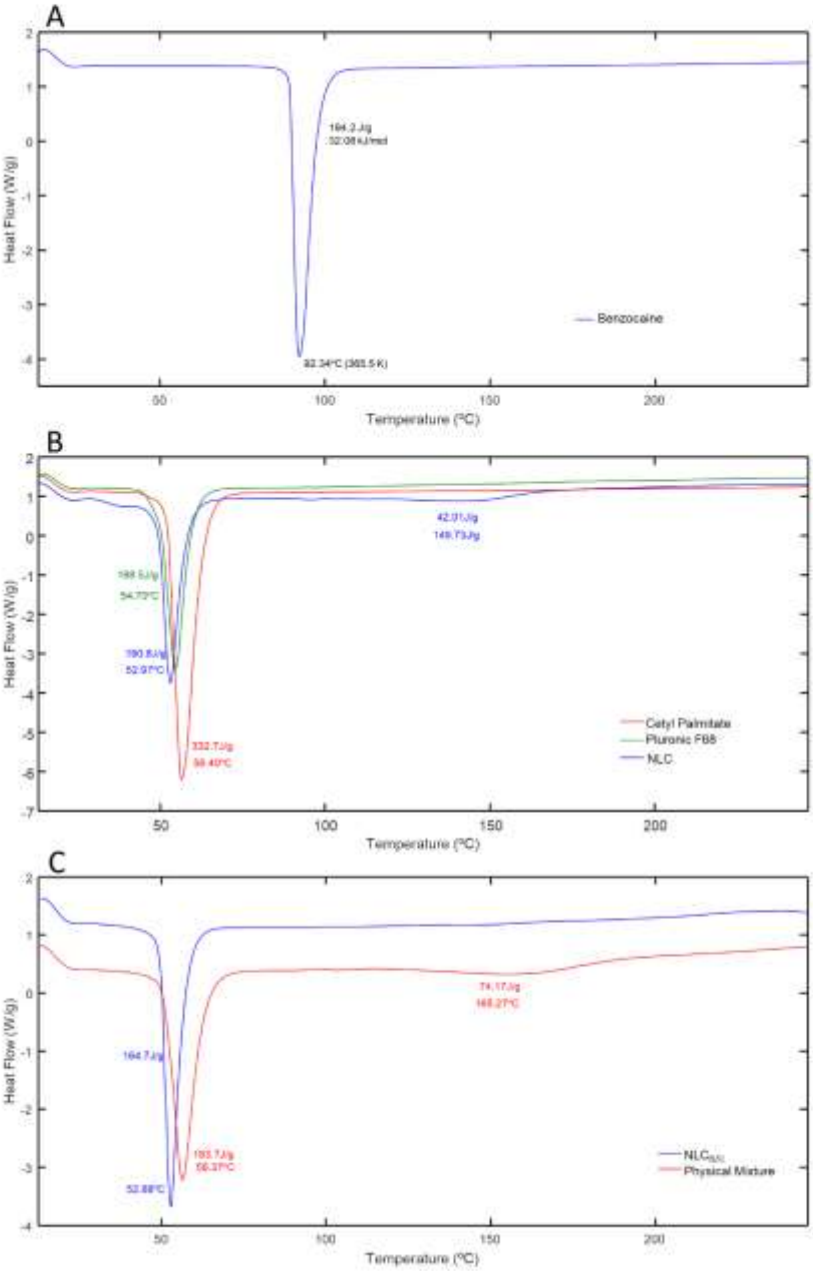

Figure S1. DSC thermograms of: A) BZC; B) CP, P68 and control NLC; C) Physical mixture and NLC_{BZC}. Heating rate of 10 °C min^{-1}.

Table S1: Preliminary tests for the preparation of NLC_{BZC}, employing different solid and liquid lipids.

**Formulation**	**A**	**B**	**C**	**D**	**E**	**F**	**G**	**H**	**I**
**Apfil®**	7%	7%	7%	-	-	-	-	-	-
**White beeswax**	-	-	-	7%	7%	7%	-	-	-
**Cetyl palmitate**	-	-	-	-	-	-	7%	7%	7%
**Labrasol®**	3%	-	-	3%	-	-	3%	-	-
**Labrafil®**	-	3%	-	-	3%	-	-	3%	-
**Propyleneglycol monocaprylate**	-	-	3%	-	-	3%	-	-	3%
Responses									
**Size (nm)**	-	-	325.3 ± 1.0	-	-	295.3 ± 1.2	-	288.3 ± 2.1	215.3 ± 0.4
**PDI**	-	-	0.356 ± 0.056	-	-	0.332 ± 0.229	-	0.432 ± 0.129	0.097 ± 0.029
**ZP |mV|**	-	-	-16.5 ± 0.1	-	-	-16.9 ± 1.0	-	-18.3 ± 0.7	-31.3 ± 0.6
**Visual aspect**	solid	solid	liquid	solid	solid	liquid	solid	liquid	liquid

Table S2. DLS: average particle size, polydispersity index (PDI) and zeta potential (mV) determined for the optimized NLC_{BZC} formulation and its control (NLC), prepared without benzocaine.

	**Size (nm)**	**PDI**	**Zeta potential |mV|**
**NLC**	180.2 ± 1.7	0.085 ± 0.022	-28.2 ± 0.5
**NLC_{BZC}**	188.1 ± 3.6	0.090 ± 0.024	-35.2 ± 0.8

Table S3. NTA: average and cumulative data of particle size. D10, D50, D90 refer to the diameter of 10%, 50% and 90% of the cumulative distribution profile of the nanoparticles. Average size, polydispersity (Span index) and number of particles/mL of the optimized (NLC_{BZC}) formulation and its control (NLC), prepared without benzocaine. n=3.

Sample	D10 (nm)	D50 (nm)	D90 (nm)	Average diameter (nm)	Span index	Particle concentration (x10^{14} NLC/mL)
**NLC**	98.7 ± 1.2	126.0 ± 1.0	179.8 ± 8.0	134.1 ± 2.9	0.64	1.67 ± 0.02
**NLC_{BZC}**	108.0 ± 0.5	142.9 ± 2.8	219.0 ± 9.7	155.0 ± 2.0	0.78	1.90 ± 0.07

Table S4: Mathematical models applied to the Release Kinetic curves of NLC and NLC_{BZC}.

	**R^2**						
**Models**	Zero order	First order	Higuchi	Hixson-Crowell	Korsmeyer-Peppas	Weibull	Baker-Lonsdale
**NLC_{BZC}**	0.9233	0.4685	0.5789	0.6814	0.8939	0.9108	0.9472
